# Supplementary figures and images for: Cloning expression and immunogenicity analysis of inhibin gene in Ye Mule Aries sheep
Source: PeerJ. 2019 Sep 25;7:e7761. doi: 10.7717/peerj.7761 (PMC6765352; doi:10.7717/peerj.7761)

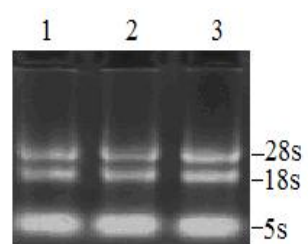

Supplement: Figure S1 — After extraction of RNA, the clear bands of RNA in 5s, 18s and 28s were detected by gel electrophoresis, which provided the assurance of quality control for the follow-up experiment. Through Fig. 1, we can see that the RNA extracted in this experiment has clear bands in all three places. The results indicated that RNA was not degraded and met the requirements of subsequent experiments. [file peerj-07-7761-s001.pdf]

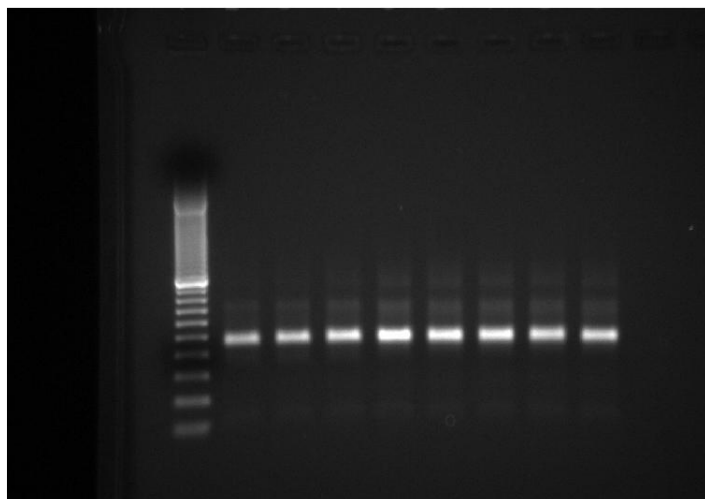

Supplement: Figure S2 — After PCR primer amplification of the cDNA sequence, it was found by gel electrophoresis that the target gene was 1,109 bp in length, as shown in Fig. 2. The picture is clear, the effect is good, and it meets the experimental requirements. [file peerj-07-7761-s002.pdf]

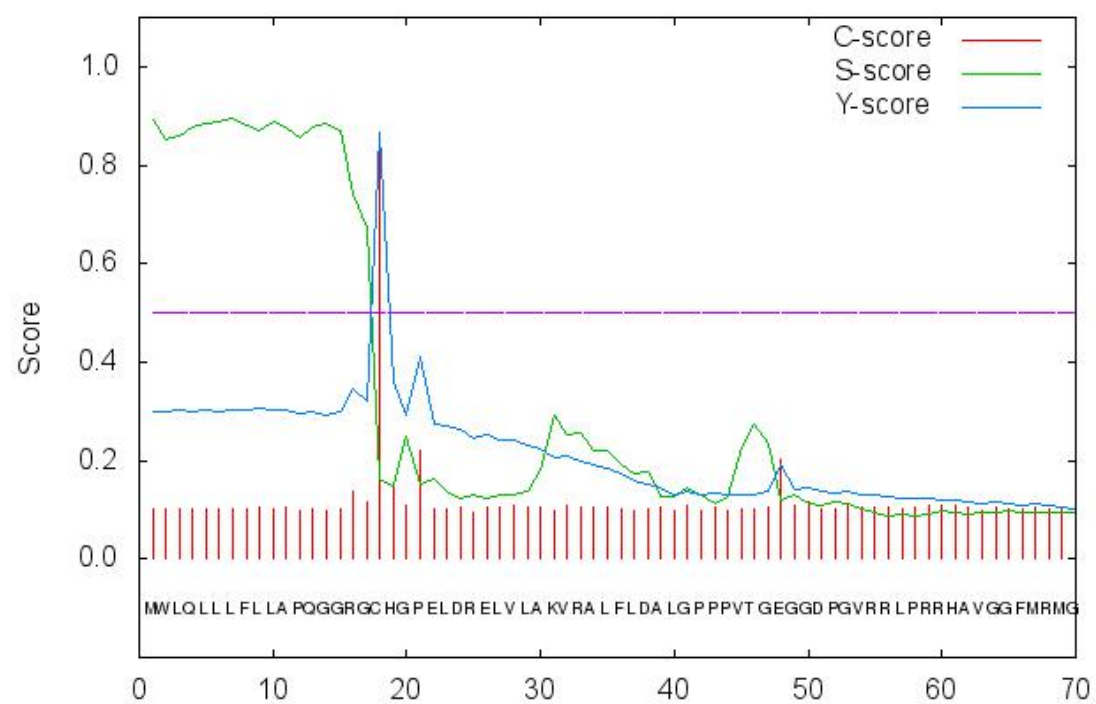

Supplement: Figure S3 — In order to analyze the bioinformatics of the cloned sequence, this study predicted by SignalP4.1 software (http://www.cbs.dtu.dk/services/SignalP/) that the Muller Aries protein was in the 17th and 18th. There is a cut point of the signal peptide between the amino acids (D = 0.861, D-cutoff = 0.450), and the whole protein includes a 17 amino acid signal peptide and a 343 amino acid mature peptide (see Figs. 2–5). Reference data is provided for conducting the WB test. [file peerj-07-7761-s003.pdf]

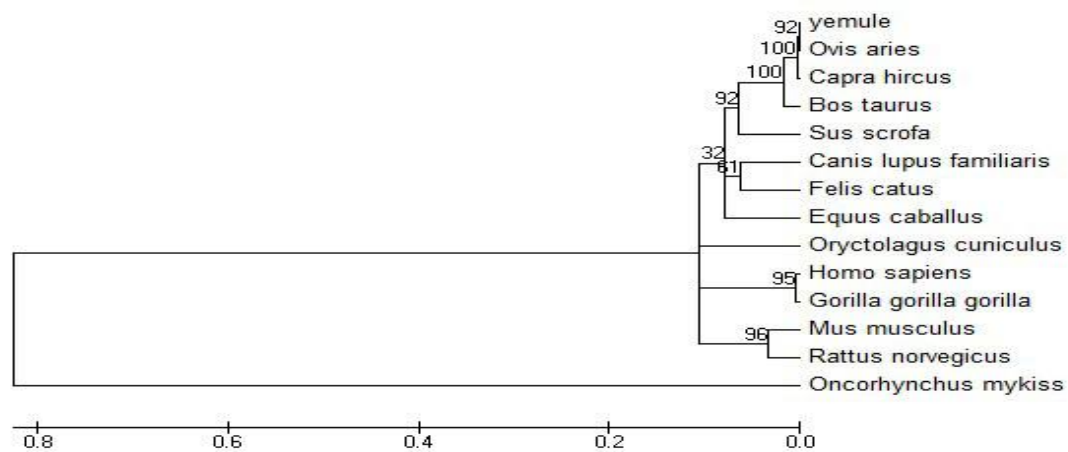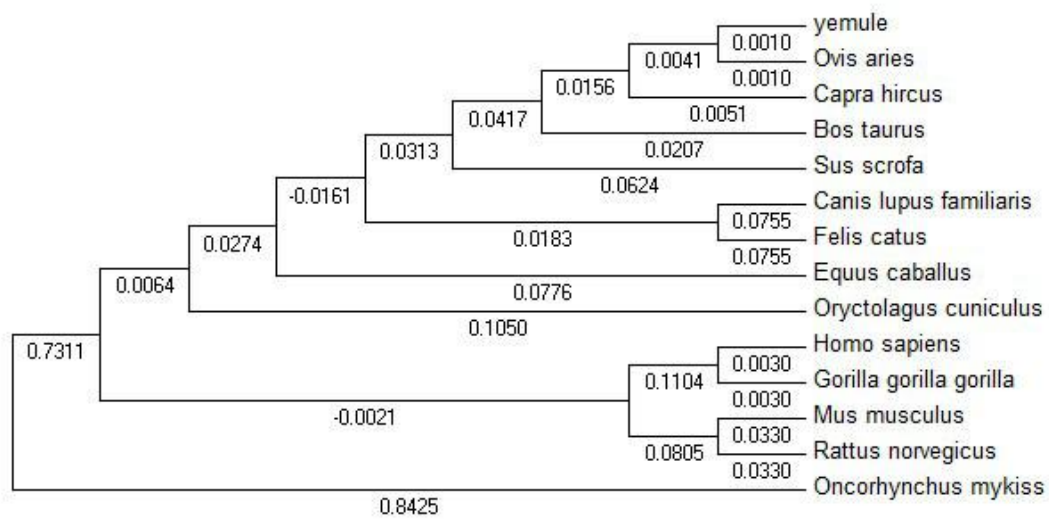

Supplement: Figure S6 — In order to understand the genetic characteristics of the INH gene, the tree analysis of the INH gene of Ye mule aries based on Mega5.0 software can clearly reflect the evolutionary genetic characteristics of organisms from aquatic to terrestrial, from lower to higher (Figs. 6). [file peerj-07-7761-s006.pdf]

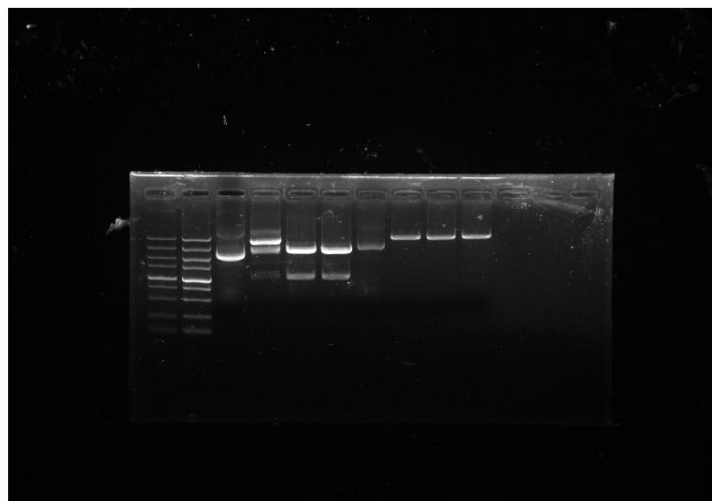

Supplement: Figure S7 — The recombinant plasmid pEGFP-INHα was digested with ScalI and EcoRI endonucleases and detected by agarose electrophoresis. It was confirmed that the INH gene was successfully inserted into the pEGFP expression vector, indicating the successful construction of the recombinant plasmid (Figs. 7). [file peerj-07-7761-s007.pdf]

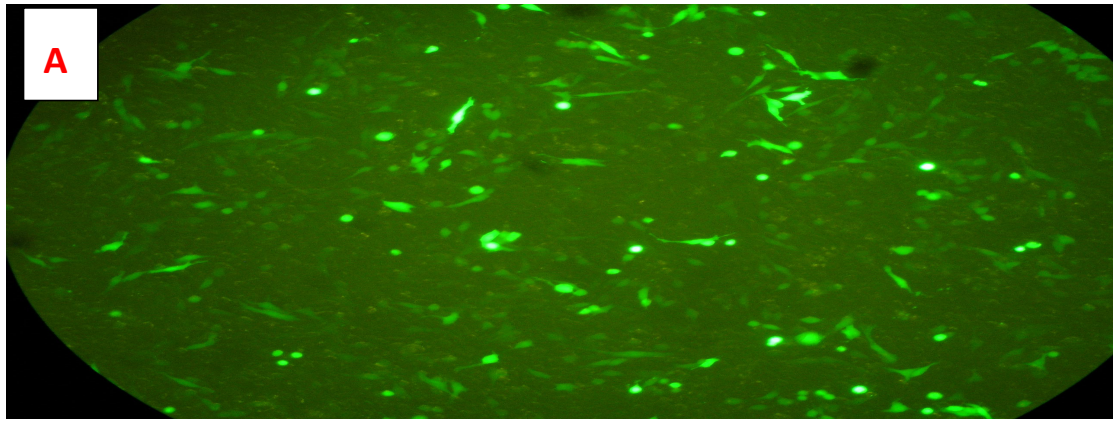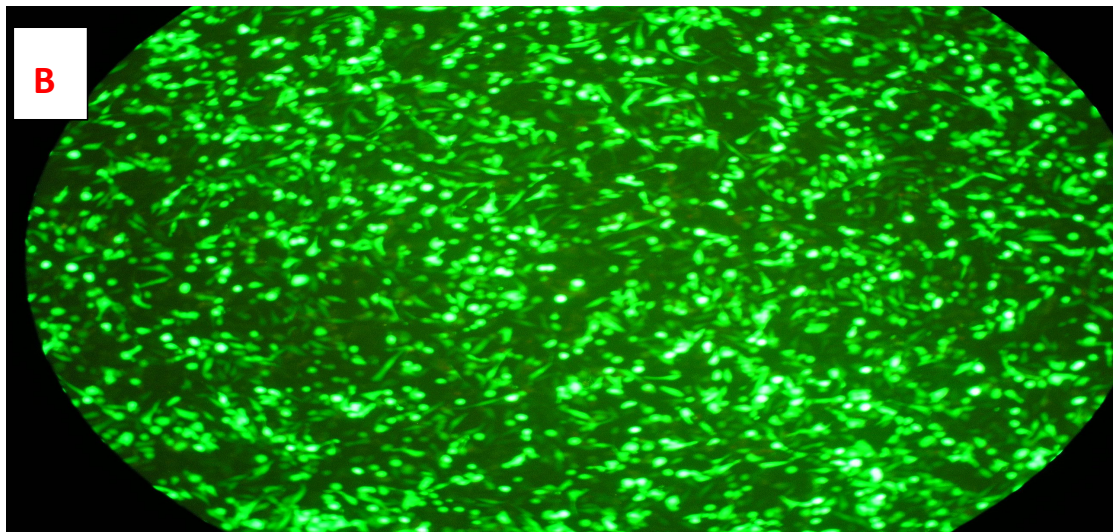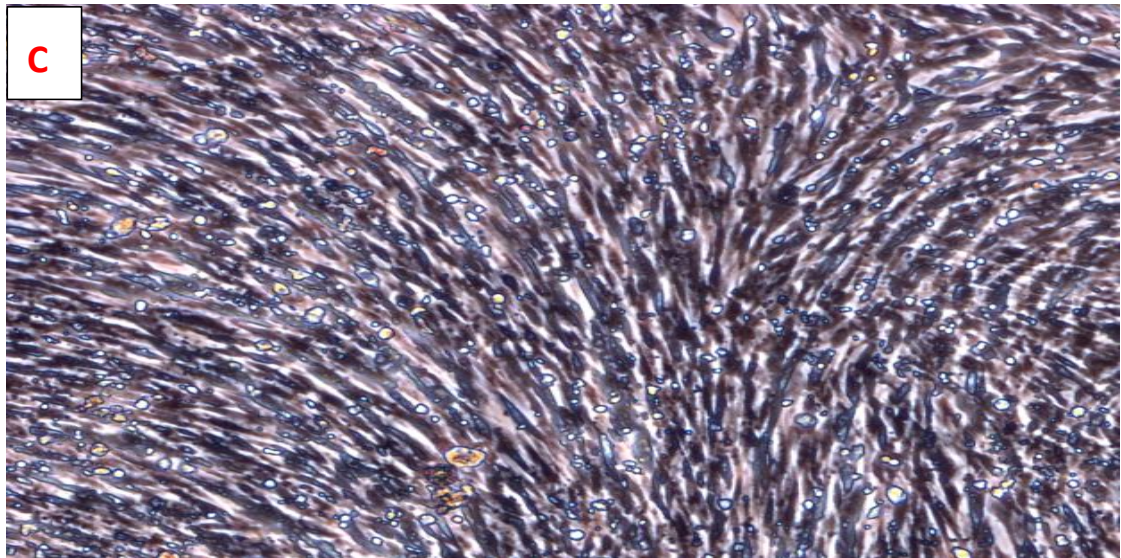

Supplement: Figure S8 — In order to analyze the expression of recombinant plasmid in cells, BHK cells were used as the research model, and the empty vector and recombinant plasmid were infected with BHK cells under the same conditions. The growth state and transfection efficiency of the cells were observed every 24 hours after infection. It was observed that the recombinant plasmid was most effective at 48 h after transfection (Figs. 8). [file peerj-07-7761-s008.pdf]

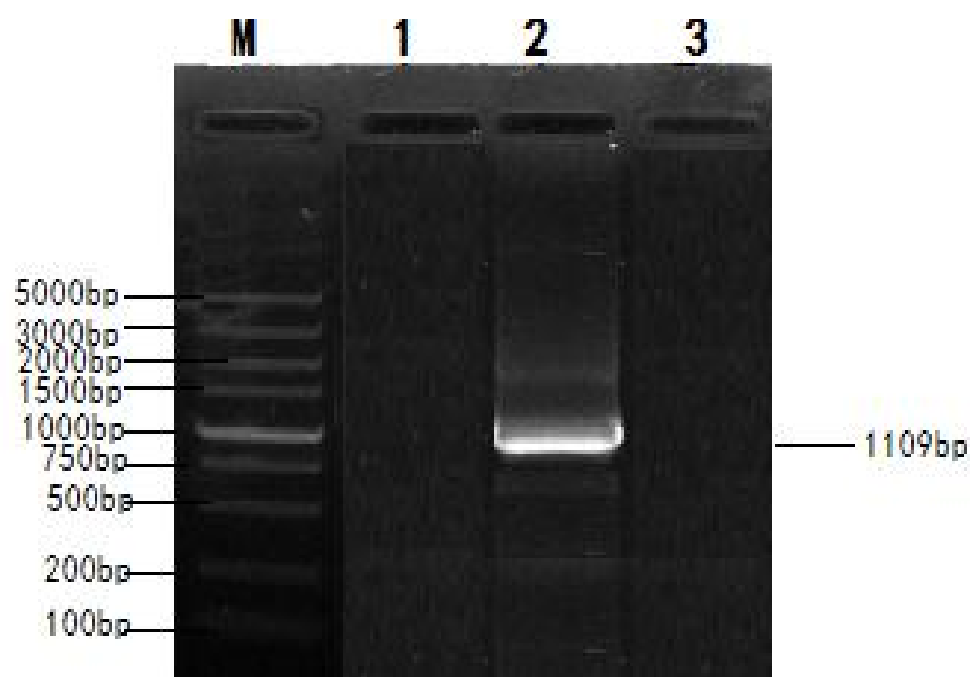

Supplement: Figure S9 — In order to analyze the expression of recombinant plasmid in cells, the expression of INH in BHK cells was detected by PCR. The results showed that the recombinant plasmid could be expressed normally in cells [file peerj-07-7761-s009.pdf]

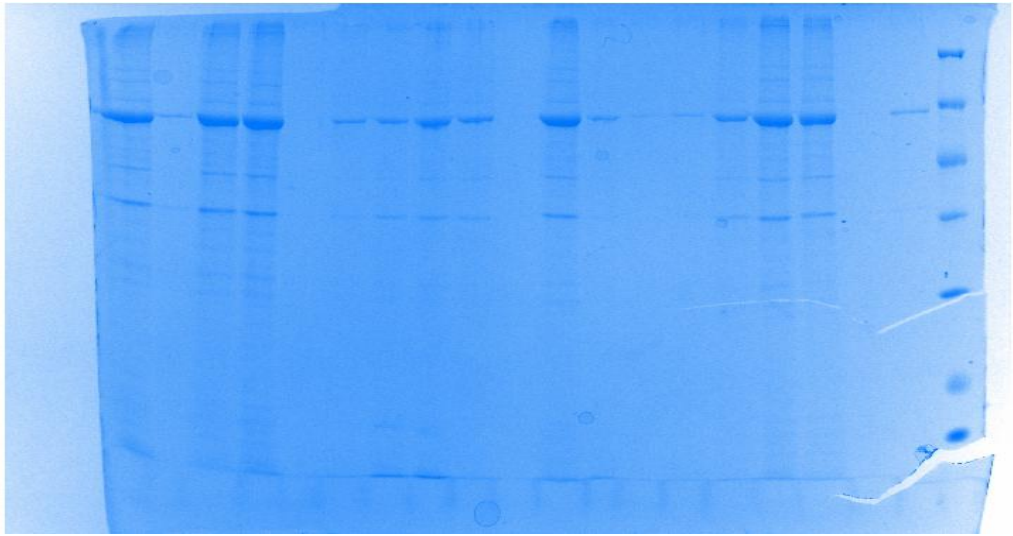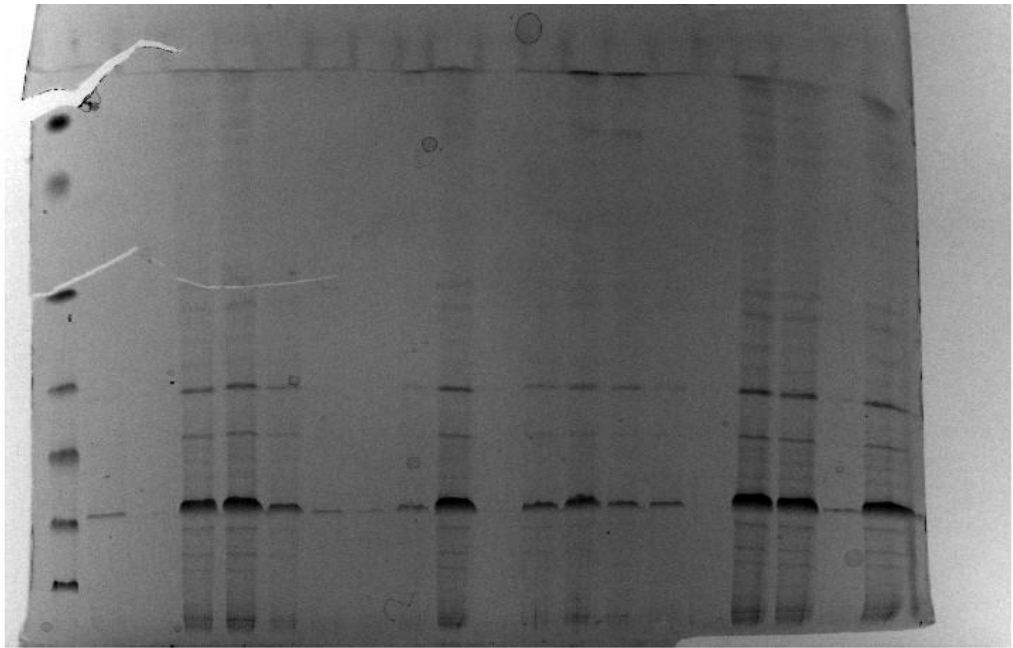

Supplement: Figure S10 — In order to further analyze the protein expression of INH gene, the protein size of INH gene expression was detected by western blotting technique was 40 KDa. [file peerj-07-7761-s010.pdf]
