# Supplementary material for: Cloning expression and immunogenicity analysis of inhibin gene in Ye Mule Aries sheep
Source: PeerJ. 2019 Sep 25;7:e7761. doi: 10.7717/peerj.7761 (PMC6765352; doi:10.7717/peerj.7761)
Supplement: Figure S4 — In order to analyze and predict the INHα protein, in this experiment, we use the NCBI database (http://www.ncbi.nlm.nih.gov/Structure/cdd/wrpsb.cgi) online tool Conserved Domain Search Service software analysis It was found that the INHα protein has one transforming growth factor TGF-beta domain at amino acids 253 to 360 and one transforming growth factor TGF-ßfamily member active domain at amino acids 256 to 360 (Fig. 4). [file peerj-07-7761-s004.pdf]

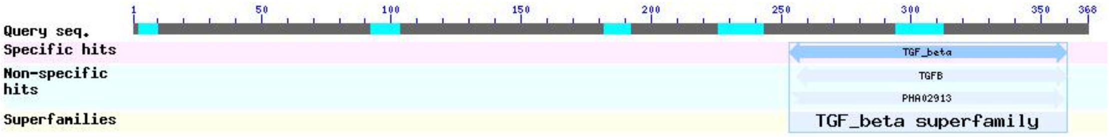

List of domain hits

Name	Accession	Description	Interval	E-value
+] TGF_beta	pfam00019	Transforming growth factor beta like domain;	253-360	9.52e-25
+] TGFB	smart00204	Transforming growth factor-beta (TGF-beta) family; Family members are active as ...	256-360	2.35e-23
+] PHA02913	PHA02913	TGF-beta-like protein; Provisional	254-359	4.31e-03
