# Supplementary material for: Cloning expression and immunogenicity analysis of inhibin gene in Ye Mule Aries sheep
Source: PeerJ. 2019 Sep 25;7:e7761. doi: 10.7717/peerj.7761 (PMC6765352; doi:10.7717/peerj.7761)
Supplement: Figure S5 — In order to predict the function of INHα gene, this experiment used DNA MAN software to analyze the homology of INH α gene of different breeds of animals, and found that the homology of INHα gene in mammals is relatively high, thus It can be concluded that the INHα gene is highly conserved (Figs. 5). [file peerj-07-7761-s005.pdf]

Percent Identity

Divergence

	1	2	3	4	5	6	7	8	9	10	11	12	13	14	15	
1		84.4	84.3	95.2	86.1	97.9	99.8	88.1	88.4	83.6	85.5	83.4	80.4	79.0	51.5	1
2	17.8		99.4	77.2	84.3	83.6	83.7	81.7	78.7	81.3	82.0	85.1	76.0	77.7	49.6	2
3	18.0	0.6		77.1	84.5	83.5	83.7	81.6	78.6	81.8	82.3	85.0	75.3	78.1	49.3	3
4	5.0	27.3	27.6		77.7	96.0	93.5	80.4	79.9	76.0	77.5	82.8	74.0	72.1	49.9	4
5	15.6	18.0	17.8	26.6		85.5	85.2	81.6	81.3	84.6	85.9	85.5	79.2	78.9	48.4	5
6	2.1	18.7	18.9	4.1	16.3		98.1	87.7	87.9	82.5	84.2	83.3	79.6	78.2	51.2	6
7	0.2	18.6	18.8	6.8	16.7	1.9		87.8	88.1	82.9	85.0	83.6	79.5	78.5	51.6	7
8	13.2	21.3	21.5	22.9	21.4	13.6	13.5		99.6	80.3	81.2	84.8	76.9	75.7	50.3	8
9	12.8	25.3	25.4	23.5	21.8	13.4	13.2	0.4		77.7	78.7	84.9	74.8	73.4	49.3	9
10	18.7	21.8	21.2	29.0	17.4	20.2	19.6	23.1	26.6		86.9	82.5	72.2	74.8	49.7	10
11	16.3	21.0	20.6	26.9	15.9	17.9	16.9	21.9	25.3	14.6		83.6	76.6	76.8	49.3	11
12	19.1	16.9	17.0	19.8	16.3	19.2	18.8	17.2	17.0	20.1	18.8		80.3	81.0	52.2	12
13	23.1	29.7	30.7	32.3	24.9	24.1	24.4	28.1	31.1	35.5	28.6	23.2		92.6	47.5	13
14	25.0	27.2	26.6	35.3	25.2	26.2	25.8	29.9	33.3	31.4	28.3	22.4	7.8		48.7	14
15	79.9	85.9	87.3	83.9	91.0	80.3	79.5	83.5	86.6	85.0	87.0	78.7	94.3	89.0		15
	1	2	3	4	5	6	7	8	9	10	11	12	13	14	15	

1、也木勒白羊yemule
2、人类Homo sapines
3、大猩猩 Gorilla
4、家牛 Bos taurus
5、家马 Equus caballus
6、山羊 Capra hircus
7、绵羊 Ovis aries
8、猪 Pig
9、野猪 Sus scrofa
10、家犬 Canis lupus
11、家猫 Felis catus
12、家兔 Oryctolagus
13、小家鼠 Mus musculus
14、褐家鼠 Rattus norvegicus
15、虹鳟鱼 Dncorhynchus mykiss
