# Supplementary material for: Cloning expression and immunogenicity analysis of inhibin gene in Ye Mule Aries sheep
Source: PeerJ. 2019 Sep 25;7:e7761. doi: 10.7717/peerj.7761 (PMC6765352; doi:10.7717/peerj.7761)
Supplement: Supplemental Information 2 — In order to study the immunogenicity of the recombinant plasmid pEGFP-INHa, the recombinant plasmid pEGFP-INHa was purified in the experiment, and the rabbit leg muscles were injected and immunized regularly according to the pre-established immunization program. Before immunization and after the first immunization 10 The changes of INH gene antibody in rabbit serum were detected on days and 20 days after the first immunization. The statistical results are shown in Table 1. [file peerj-07-7761-s012.pdf]

Changes of INH after immunization in experimental rabbits

Order \ Date	0 d		10 d		20 d	
	Immunization group	negative control	Immunization group	negative control	Immunization group	negative control
**A**	0.088	0.09	0.761	0.11	1.35	0.16
**B**	0.092	0.091	0.739	0.12	1.13	0.1
**C**	0.088	0.091	0.576	0.08	1.41	0.08
**D**	0.092	0.09	0.56	0.07	1.24	0.13
**E**	0.087	0.09	0.755	0.09	1.05	0.13
**F**	0.093	0.088	0.97	0.08	1.3	0.07
**G**	0.092	0.09	0.71	0.09	1.12	0.13
**H**	0.088	0.092	0.94	0.08	1.24	0.086
