# Supplementary material for: Cloning expression and immunogenicity analysis of inhibin gene in Ye Mule Aries sheep
Source: PeerJ. 2019 Sep 25;7:e7761. doi: 10.7717/peerj.7761 (PMC6765352; doi:10.7717/peerj.7761)
Supplement: Supplemental Information 3 — In order to further study the regulation of INH on FSH, rabbits were used as experimental subjects in this experiment. The recombinant plasmid was immunized with leg muscle according to the pre-designed immune program, and was immunized 10 days before immunization and 20 days after immunization. The changes of FSH in rabbit serum were shown in Table 2. [file peerj-07-7761-s013.pdf]

Changes of FSH after immunization in experimental rabbits

Date / Order	0 d		10 d		20 d	
	Immunization group	negative control	Immunization group	negative control	Immunization group	negative control
**A**	1.10	0.84	2.35	1.47	3.11	1.24
**B**	1.52	1.85	1.81	1.39	2.47	1.62
**C**	1.68	0.82	1.20	1.32	2.07	1.47
**D**	1.36	1.17	1.54	1.37	2.64	1.38
**E**	0.35	1.59	2.11	0.97	2.79	1.10
**F**	1.36	1.55	1.39	1.72	3.09	1.31
**G**	1.33	1.81	1.56	1.61	2.80	1.25
**H**	1.86	1.58	1.45	1.25	2.50	1.43
